# A Novel Speed Compensation Method for ISAR Imaging with Low SNR

**DOI:** 10.3390/s150818402

**Published:** 2015-07-28

**Authors:** Yongxiang Liu, Shuanghui Zhang, Dekang Zhu, Xiang Li

**Affiliations:** College of Electronic Science and Engineering, National University of Defense Technology, Changsha 410073, China; E-Mails: zhangshuanghui@nudt.edu.cn (S.Z.); dekang.zhu@foxmail.com (D.Z.); lixiang01@vip.sina.com (X.L.)

**Keywords:** ISAR, radar imaging, SNR, cubic phase function

## Abstract

In this paper, two novel speed compensation algorithms for ISAR imaging under a low signal-to-noise ratio (SNR) condition have been proposed, which are based on the cubic phase function (CPF) and the integrated cubic phase function (ICPF), respectively. These two algorithms can estimate the speed of the target from the wideband radar echo directly, which breaks the limitation of speed measuring in a radar system. With the utilization of non-coherent accumulation, the ICPF-based speed compensation algorithm is robust to noise and can meet the requirement of speed compensation for ISAR imaging under a low SNR condition. Moreover, a fast searching implementation strategy, which consists of coarse search and precise search, has been introduced to decrease the computational burden of speed compensation based on CPF and ICPF. Experimental results based on radar data validate the effectiveness of the proposed algorithms.

## 1. Introduction

Radar is emerging as an outstanding tool for the acquisition of a target at long range, in all weather, in day and night circumstances. Inverse synthetic aperture radar (ISAR) imaging is an active imaging technology, which can provide a two-dimensional high-resolution image of the moving target. It has been widely applied in many civil and military fields, such as air control, air defense, space surveillance, missile defense, and so on [1,2,3].

Since ISAR imaging is mainly applied to image non-cooperative moving targets (aircraft, warships, missiles, satellites, *etc*.), the quality of the images is mainly determined by the performance of motion compensation of the radar echoes. In general, speed compensation is the first step of motion compensation for ISAR imaging, which is developed to eliminate the effect of speed on the range profiles. For targets with a low speed, a well-focused ISAR image can be achieved by the assumption of the “go-stop” model, in which the targets can be considered as stationary during the interval of one radar transmitted pulse. For a target with high speed, however, the assumption of the “go-stop” model is limited and will defocus the range profiles and ISAR image of the target. Moreover, the low SNR condition in the radar echoes often challenges the performance of the speed compensation for ISAR imaging, and the factors inducing low SNR include the long distance between radar and target, the small size of the radar cross-section (RCS) of the target, and so on. Hence, the research on high speed compensation for ISAR imaging under low SNR conditions is of great academic significance for ISAR imaging radar systems and applications.

As far as the radar system for speed compensation is concerned, the ISAR system usually is accompanied by the narrowband mode, which conducts the functions of searching and tracking the target. This radar system mode rarely provides high enough precision for the target speed for ISAR imaging, while inducing implementation difficulties in the radar system. An alternative solution is to estimate speed directly from the wideband radar echo by signal processing, which has the advantage of decreasing the burden of the radar system, and this work has attracted much attention in theradar community.

Several speed compensation algorithms for ISAR imaging have been proposed in the past few years, such as random-ambiguity transform (RAT) [4], discrete chirp-Fourier transform (DCFT) [5], discrete match Fourier transform (DMFT) [6], the product high-order ambiguity function (PHAF) [7], adaptive joint time-frequency representation based on particle swarm optimization (PSO-AJFT) [8], fractional Fourier transform (FRFT) [9], and so on. As far as the performances of these algorithms are concerned, RAT suffers from heavy computational burden due to the polar coordinate interpolation. DCFT, DMFT, PHAF and PSO-AJFT have higher computational efficiency, but lower precision. FRFT can obtain the high-resolution range profile (HRRP) of the target without estimating the speed, but its performance is sensitive to noise and can hardly satisfy the requirement of ISAR imaging in low SNR conditions.

In this paper, the cubic phase function (CPF) [10,11] and the integrated cubic phasefunction (ICPF) [12] are introduced to speed compensation for ISAR imaging. ICPF is developed from CPF and generally applied to estimate the frequency modulation rate of the multi-component linear frequency-modulated (LFM) signal. ICPF can improve SNR and suppress the cross-terms with the process of integration, which ensures the robustness of the speed compensation based on ICPF. Experimental results based on the radar data of a cone-shaped model and an aircraft validate the effectiveness of the proposed algorithms.

The paper is organized as follows: the radar echo model of speed compensation is presented in Section 2; the speed compensation methods based on CPF and ICPF are proposed in Section 3; experimental results based on radar data are illuminated in Section 4; and conclusions are summarized in Section 5.

## 2. Radar Echo Model of Speed Compensation

The transmitted LFM signal is as follows:

(1) $$s\left(\hat{t},t_{m}\right)=rect\left(\frac{\hat{t}}{T_{p}}\right)\text{exp}\left[j2\pi \left(f_{c}t+\frac{\gamma }{2}{\hat{t}}^{2}\right)\right]$$

where $t_{m}$, $\hat{t}$, $f_{c}$, $T_{p}$ and *γ* represent the slow time, fast time, center frequency, pulse width and frequency modulation rate, respectively, and $t=\hat{t}+t_{m}$. rect(·) is a window function defined as:

(2) $$rect\left(u\right)=\left\{\begin{array}{cc} 1 & \left|u\right|\leq \frac{1}{2}\\ 0 & |u|>\frac{1}{2} \end{array}\right.$$

Then, the radar echo can be represented as follows:

(3) $$s_{r}\left(\hat{t},t_{m}\right)=A\cdot rect\left(\frac{\hat{t}-\frac{2R}{c}}{T_{p}}\right)\text{exp}\left\{j2\pi \left[f_{c}\left(t-\frac{2R}{c}\right)+\frac{\gamma }{2}{\left(\hat{t}-\frac{2R}{c}\right)}^{2}\right]\right\}$$

where *R* is the range between radar and target.

The process of de-chirping is usually used on the radar echo to decrease the burden of the radar receiver, in which the reference signal used is as follows:

(4) $$s_{ref}\left(\hat{t},t_{m}\right)=A\cdot rect\left(\frac{\hat{t}-\frac{2R_{ref}}{c}}{T_{ref}}\right)\text{exp}\left\{j2\pi \left[f_{c}\left(t-\frac{2R_{ref}}{c}\right)+\frac{\gamma }{2}{\left(\hat{t}-\frac{2R_{ref}}{c}\right)}^{2}\right]\right\}$$

where $R_{ref}$ and $T_{ref}$ are the reference range and pulse width, respectively.

Then, the process of de-chirping can be achieved as follows:

(5) $$\begin{array}{cc} s_{de}\left(\hat{t},t_{m}\right) & =s_{r}\left(\hat{t},t_{m}\right)\cdot s_{ref}^{*}\left(\hat{t},t_{m}\right)\\ & =A\cdot rect\left(\frac{\hat{t}-\frac{2R}{c}}{T_{p}}\right)\text{exp}\left[-j\gamma \frac{4\pi }{c}R_{\Delta }\left(\hat{t}-\frac{2R_{ref}}{c}\right)-j\frac{4\pi }{c}f_{c}R_{\Delta }+j\frac{4\pi \gamma }{c^{2}}R_{\Delta }^{2}\right] \end{array}$$

where $R_{\Delta }=R-R_{ref}$. As shown in Equation (5), there are three phase terms in the de-chirped signal, in which the first and second ones are necessary for achieving down-range and cross-range resolution, respectively, and the third one is the residual video phase (RVP), which can be compensated by the process of de-ramping [13]. Note that the de-chirped signal is a single-frequency signal with respect to the fast time $\hat{t}$; HRRP of the target can be obtained by applying fast Fourier transform (FFT) to the de-chirped signal along the fast time. For a high speed target, however, the range between radar and target will vary as the fast time. Suppose that the target moves at a stable speed within one pulse. Then, the range between radar and target can be represented as $R=R_{m}+v\hat{t}$, where $R_{m}$ only varies as the slow time. The $R_{\Delta }$ shown in Equation (5) can be rewritten as:

(6) $$R_{\Delta }=R_{\Delta }^{\prime }+v\hat{t}$$

where $R_{\Delta }^{\prime }=R_{m}-R_{ref}$. Substitute Equation (6) into Equation (5):

(7) $$s_{d}\left(\hat{t},t_{m}\right)=A\cdot rect\left(\frac{\hat{t}-\frac{2R_{m}}{c}-\frac{2v\hat{t}}{c}}{T_{p}}\right)\text{exp}\left(j2\pi \left(\varphi_{1}+\varphi_{2}\hat{t}+\varphi_{3}{\hat{t}}^{2}\right)\right)$$

where:

(8) $$\begin{array}{cc} & \varphi_{1}=-\frac{2}{c}\left(-\frac{2\gamma }{c}R_{ref}R_{\Delta }^{\prime }+f_{c}R_{\Delta }^{\prime }-\frac{\gamma }{c}R_{\Delta }^{\prime 2}\right)\\ & \varphi_{2}=-\frac{2}{c}\left(\gamma R_{\Delta }^{\prime }-\frac{2\gamma }{c}R_{ref}v+f_{c}v-\frac{2\gamma }{c}R_{\Delta }^{\prime }v\right)\\ & \varphi_{3}=-\frac{2\gamma v}{c}\left(1-\frac{v}{c}\right) \end{array}$$

As shown in Equation (7), the de-chirped signal is changed to be an LFM signal because of the effect of the speed of the target, and $\varphi_{1}$, $\varphi_{2}$ and $\varphi_{3}$ are the constant, linear and quadratic coefficients of the LFM signal, respectively. Each term is analyzed in the ensuing paragraphs.

There are three parts in the term $\varphi_{1}$, in which the first part is introduced by $R_{ref}$ and can be eliminated by FFT [12], and the second and third parts are the Doppler frequency term and the RVP, respectively. The term $\varphi_{2}$ consists of four parts, where the first part provides down-range resolution; the second part is also introduced by $R_{ref}$; the third part shifts the range profile and should be compensated by speed compensation; and since $\frac{v}{c}\approx 0$, the fourth part can be ignored when compared to the first part. As far as the term $\varphi_{3}$ is concerned, it is approximately equal to $-\frac{2\gamma v}{c}{\hat{t}}^{2}$ because of $\frac{v}{c}\approx 0$, which de-focuses the range profile and also needs to be compensated. To summarize, the residual phase to be compensated is as follows:

(9) $$p\left(v\right)=\text{exp}\left[-j4\pi v\left(f_{c}\hat{t}+\gamma {\hat{t}}^{2}\right)\right]$$

It can be seen from Equation (7) that the speed of the target can be achieved by estimating the quadratic coefficient of the de-chirped signal. CPF [9,10] and ICPF [11] are two novel methods to estimate the instantaneous frequency rate (IFR) of the noisy quadratic frequency-modulated (QFM) signal. In the next section, we introduce CPF and ICPF to the estimation and compensation of speed in a lowSNR condition.

## 3. Speed Compensation Based on CPF and ICPF

### 3.1. CPF

CPF has been proposed [10] to estimate the IFR of the noisy QFM. For the noisy LFM, CPF can also be used to estimate the frequency modulation rate of the LFM signal. Suppose that the LFM signal submerged in Gaussian white noise is as follows:

(10) $$x\left(n\right)=s\left(n\right)+v\left(n\right)=A\text{exp}\left(j(a_{0}+a_{1}n+a_{2}n^{2})\right)+v\left(n\right)$$

where $n=-\frac{N-1}{2},-\frac{N-1}{2}+1,\cdots ,\frac{N-1}{2}$ and v(n) is the additive white Gaussian noise with a mean of zero. Then, transform x(n) as follows:

(11) $$\begin{array}{cc} & x\left(n+m\right)x\left(n-m\right)=A^{2}\text{exp}\left\{j2\left[\left(a_{0}+a_{1}n+a_{2}n^{2}\right)+a_{2}m^{2}\right]\right\}\\ & +Av\left(n-m\right)\text{exp}\left\{j\left[a_{0}+a_{1}\left(n+m\right)+a_{2}{\left(n+m\right)}^{2}\right]\right\}\\ & +Av\left(n+m\right)\text{exp}\left\{j\left[a_{0}+a_{1}\left(n-m\right)+a_{2}{\left(n-m\right)}^{2}\right]\right\}\\ & +v(n+m)v(n-m) \end{array}$$

Note that v(n-m) and v(n+m) are random noise, and x(n+m)x(n-m) can be seen as a quadratic phase signal with respect to *m*. Therefore, the quadratic coefficient of x(n+m)x(n-m) can be estimated by the operator $\sum_{m=0}^{\frac{N-1}{2}}\left(\cdot \right)\text{exp}\left(-j\Omega m^{2}\right)$, which is shown as follows:

(12) $$CPF_{x}\left(n,\Omega \right)=\sum_{m=0}^{\frac{N-1}{2}}x\left(n+m\right)x\left(n-m\right)\text{exp}\left(-j\Omega m^{2}\right)$$

Then, the frequency modulation rate of s(n) can be estimated as:

(13) $${\hat{a}}_{2}=\text{arg}\;\underset{\Omega }{\text{max}}\left\{|CPF_{x}\left(n_{0},\Omega \right)|\right\}/2$$

where $n_{0}\in \left\{-\frac{N-1}{2},-\frac{N-1}{2}+1,\cdots ,\frac{N-1}{2}\right\}$, which means that any slice of $CPF_{x}\left(n,\Omega \right)$ can be used to estimate $a_{2}$. For the de-chirped signal shown as Equation (7), its discrete form is as follows:

(14) $$\begin{array}{cc} & s_{d}\left(n,m\right)=A\cdot rect\left(\frac{n}{N}-\frac{2R_{m}}{cT_{p}}-\frac{2nv}{Nc}\right)\\ & \text{exp}\left\{-j\frac{4\pi }{c}\left[f_{c}R_{\Delta }^{\prime }+\frac{T_{p}}{N}\left(\gamma R_{\Delta }^{\prime }+f_{c}v\right)n+\gamma v\frac{T_{p}^{2}}{N^{2}}n^{2}\right]\right\} \end{array}$$

where *N* is the number of samples within one pulse, and the RVP term shown in Equation (7) has been eliminated for the simplicity of exposition. According to Equation (12), the CPF of $s_{d}\left(n,m\right)$ can be achieved as:

(15) $$CPF_{s_{d}}\left(n,\Omega \right)=\sum_{k=0}^{\frac{N-1}{2}}s_{d}\left(n+k,m_{0}\right)s_{d}\left(n-k,m_{0}\right)\text{exp}\left(-j\Omega k^{2}\right)$$

where $m_{0}\in \left\{0,1,\cdots ,M-1\right\}$. According to (14), the quadratic coefficient of $s_{d}\left(n,m\right)$ is as follows:

(16) $$a_{2}=-\frac{4\pi \gamma vT_{p}^{2}}{cN^{2}}$$

Combine Equation (13) with Equation (16), and the estimation of speed can be achieved as:

(17) $$\hat{v}=-\frac{cN^{2}}{2\pi \gamma T_{p}^{2}}\cdot \underset{\Omega }{\text{max}}\left\{CPF_{s_{d}}\left(n_{0},\Omega \right)\right\}$$

Then, the residual phase caused by speed in $s_{d}\left(n,m\right)$ can be compensated as:

(18) $$\begin{array}{cc} s_{d}^{c}\left(n,m\right)=s_{d}\left(n,m\right)\cdot \text{exp}\left\{j\frac{4\pi }{c}\left[\frac{f_{c}\hat{v}T_{p}}{N}n+\frac{\gamma \hat{v}T_{p}^{2}}{N^{2}}n^{2}\right]\right\} & \\ m=0,1,\cdots ,M-1 \end{array}$$

### 3.2. ICPF

Since the CPF defined as Equation (12) is quadratic, it will suffer from the effect of cross-terms when the signal is multi-component. It is shown in [12] that the auto-terms of the CPF of the LFM signal are distributed over the straight lines parallel to the time axis, while the cross-terms fluctuate with time. Hence, the integration of CPF along the time axis can enhance the energy of the auto-terms and suppress the energy of the cross-terms, which is just the definition of the ICPF. It can be represented as follows:

(19) $$ICPF_{x}\left(\Omega \right)=\sum_{n}{\left|CPF_{x}\left(n,\Omega \right)\right|}^{2}$$

Substitute Equation (15) into Equation (19):

(20) $$\begin{array}{cc} ICPF_{x}\left(\Omega \right) & =\sum_{n}\sum_{k}\sum_{l}x\left(n+k\right)x\left(n_{k}\right)\\ & =x^{*}\left(n+l\right)x*\left(n-l\right)\text{exp}\left[-j\Omega (k^{2}-l^{2})\right] \end{array}$$

ICPF of the single component LFM signal exhibits a peak at $\Omega =2a_{2}$. Additionally, for the ICPF of the multicomponent LFM signal, the peaks of auto-terms are enhanced, and those of cross-terms are suppressed. Hence, the estimation of the quadratic coefficient can be estimated by one-dimensional search of ICPF, which is shown as follows:

(21) $${\hat{a}}_{2}=\text{arg}\;\underset{\Omega }{\text{max}}\left\{|ICPF_{x}\left(\Omega \right)|\right\}/2$$

With the application of the integration operator $\sum_{n}{\left|\;\cdot \;\right|}^{2}$ shown in Equation (19), the parameter estimation based on ICPF can improve the SNR of the signal, so it still performs well in a low SNR condition.

Noting that the target generally contains several scatterers, the radar echo is always multi-component. The precision of the speed estimation based on CPF could be decreased by the effect of cross-terms. Therefore, ICPF is introduced to speed estimation to suppress cross-terms and improve SNR.

Moreover, compared to the translational movement of the target, relative movement (precession, flip, spin, *etc*.) between different scatterers is small enough to be ignored. Additionally, since the frequency modulation rates of the de-chirped radar echo from all scatterers are introduced by the speed of the target, they can be seen as the same. Therefore, the speed of the target can be estimated by one-dimensional search of ICPF of the de-chirped radar echo, which is shown as follows:

(22) $$\hat{v}=-\frac{cN^{2}}{2\pi \gamma T_{p}^{2}}\underset{\Omega }{\cdot \text{max}}\left\{ICPF_{s_{d}}\left(\Omega \right)\right\}$$

Then, the speed compensation can further be achieved by Equation (18). In the next subsection, the flow chart of the speed compensation based on CPF and ICPF is presented.

### 3.3. Flow Chart

The speed compensation based on CPF and ICPF can be achieved as follows.

(1) Initialize the bound and sample number of the search of speed, which are represented as $v_{\text{max}}$ and *K*, respectively;
(2) Calculate CPF or ICPF of the de-chirped radar echo, where the bound of the frequency axis of CPF or ICPF is determined by $v_{\text{max}}$. The relationship between $\Omega_{\text{max}}$ $v_{\text{max}}$ is shown as follows:
  (23) $$\Omega_{\text{max}}=-\frac{2\pi \gamma T_{p}^{2}}{cN^{2}}v_{\text{max}}$$
(3) Search CPF or ICPF to obtain the coarse estimation of speed by Equation (17) or Equation (22). The precision of the coarse estimation of speed is $\frac{2v_{\text{max}}}{N}$.
(4) Reduce the bound of the search to $\left[v_{c}-2\frac{v_{\text{max}}}{K},v_{\text{max}}-2\frac{v_{\text{max}}}{K}\right]$, so as to obtain more precise estimation of speed.
(5) Search CPF or ICPF to obtain the precise estimation of the speed by Equation (17) or Equation (22).
(6) Judge whether the estimation of speed reaches the expected precision. If it does, compensate the speed by Equation (18) or turn to Step Equation (4) to repeat the precise estimation.

**Figure 1:**
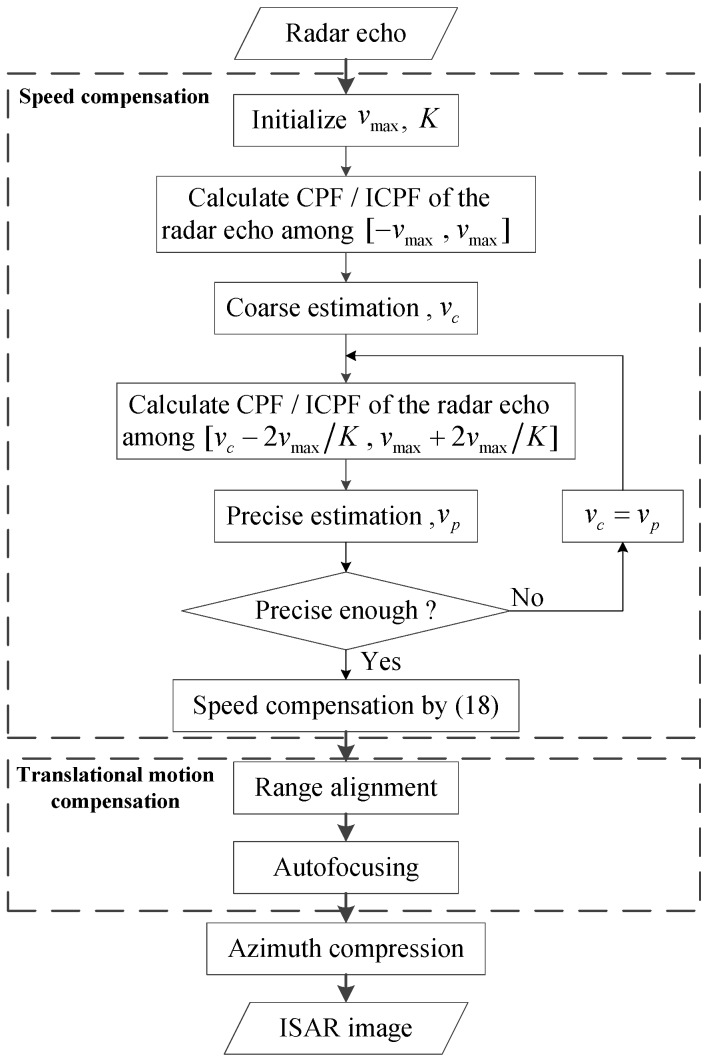
Flow chart.

**Figure 2:**
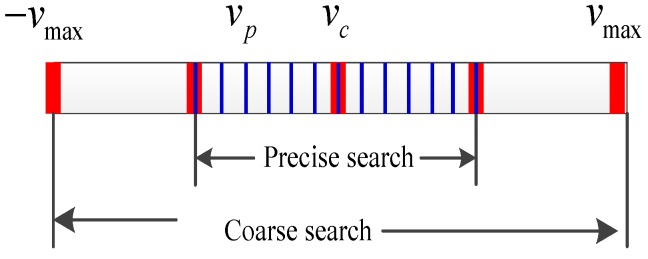
Illustration of the process of searching.

Figure 1 shows the flow chart of the estimation of the speed, and Figure 2 illustrates the coarse and precise search.

## 4. Experiments

The data of a cone-shaped model measured in the microwave chamber and the radar data of an aircraft are utilized to validate the performance of the proposed method, respectively.

### 4.1. Experiment Based on the Data of a Cone-Shaped Model

A cone-shaped model is measured by an X band radar in the microwave chamber. The waveform parameters of the radar are as follow: the center frequency is 10 GHz; the band width is 2 GHz; the pulse width is 100 *μ*s; and the pulse repetition frequency (PRF) is 200 Hz, respectively.

**Figure 3:**
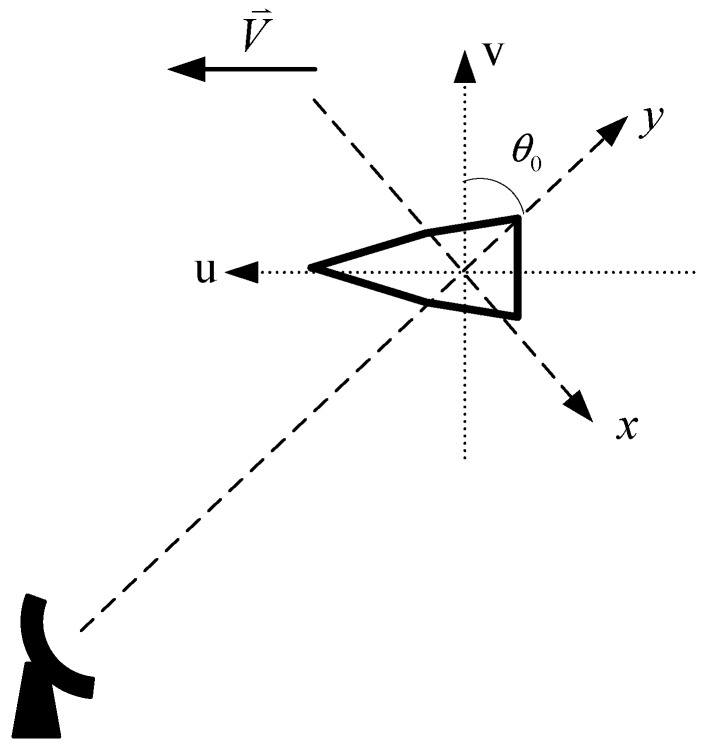
Moving scene of the target.

The motion model of the cone-shaped target is designed to compound the radar data to simulate a high speed moving target (such as a missile, satellite, *etc*.), as shown in Figure 3. In this case, the target is flying towards the radar with a speed of $\bar{V}$. The aspect angle of the target corresponding to the movement of the target is initialized as $\frac{\pi }{4}$, *i.e*., $\theta_{0}=\frac{\pi }{4}$, and it is set to be uniformly changed with a rotational speed of 0.4 rad/s.

**Figure 4:**
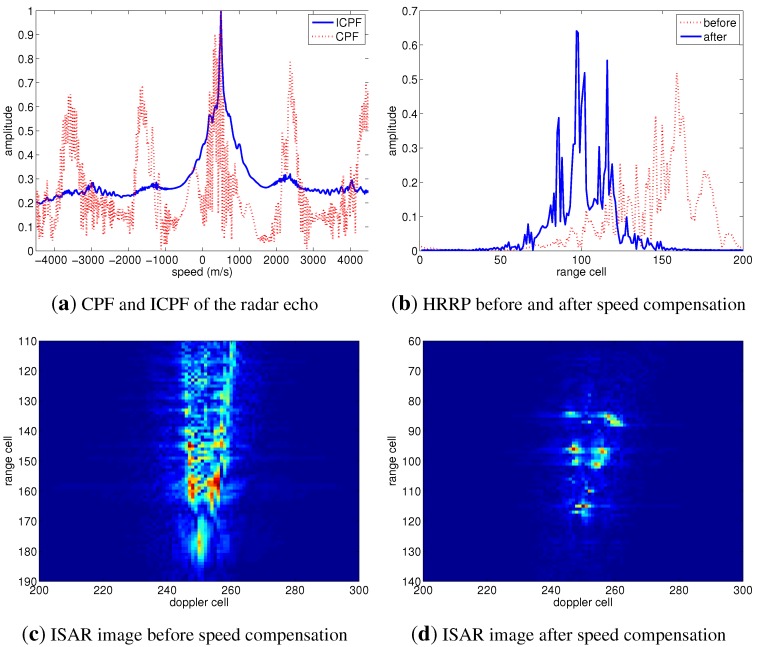
Speed compensation results of the simulated radar target with a speed of 500 m/s. CPF, cubic phase function; ICPF, inverse CPF; HRRP, high-resolution range profile.

Firstly, the target speed is set as 500 m/s. The HRRP and ISAR image of the target before speed compensation are shown as the dashed line in Figure 4b,c, respectively. It has been shown that the HRRP and the ISAR image of the target are defocused seriously due to the effect of speed, and the scatterers of the target can hardly be recognized. The proposed methods based on CPF and ICPF are utilized for speed compensation. Figure 4a shows the CPF and ICPF of the radar echo, both CPF and ICPF exhibiting a peak at 500 m/s, and the speed of the target can be estimated by one-dimensional search of the curves of CPF or ICPF. Moreover, the noise and cross-terms have been largely suppressed in ICPF, which can validate its robustness. The real line shown in Figure 4b is the HRRP after speed compensation, which is much better focused than the one without compensation, shown as the dashed line. Figure 4d shows the ISAR image after speed compensation, Comparing to the uncompensated one in Figure 4c, it is more focused, and the scatters can be recognized easily, which validates the effectiveness of the proposed algorithm.

**Figure 5:**
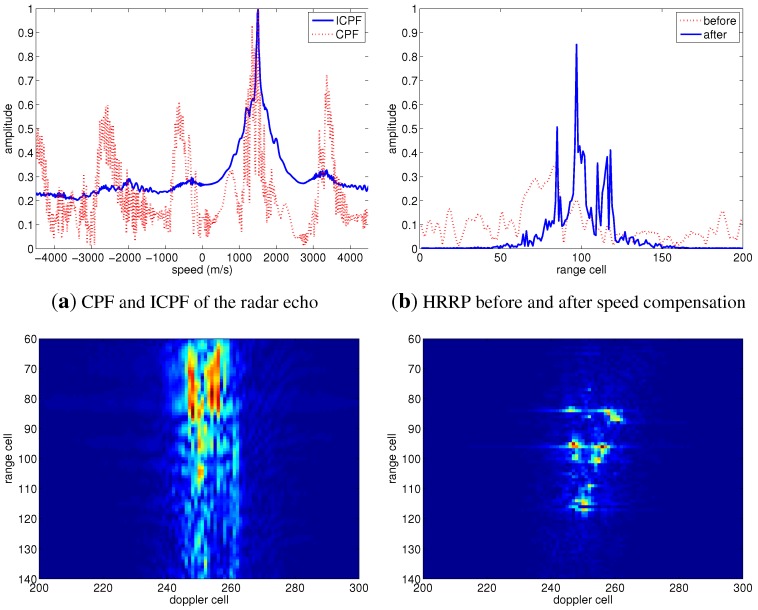
Speed compensation results of the simulated radar target with a speed of 1500 m/s.

Further, the speed of the target is set as 1500 m/s to validate the performance of the proposed methods under a high speed condition, and the experimental results are shown as Figure 5. Comparing with Figure 4b, the HRRP shown in Figure 5b is more defocused, *i.e*., the scatterers spread along range cells more obviously. The ISAR image under a speed of 1500 m/s shown in Figure 5c is more blurred than that at 500 m/s, shown as Figure 4c, which indicates that a higher target speed induces the ISAR image to be more defocused. As far as the speed estimation performance of the proposed algorithm is concerned, the result in Figure 5a shows that the curves of the CPF and ICPF of the radar echo can exhibit a peak at 1500 m/s, which means that the target speed can be estimated correctly by one-dimensional search of CPF or ICPF. After the compensation of the estimated speed, the HRRP and the ISAR image are much better focused, as shown in Figure 5b,d, respectively, demonstrating that the proposed methods are effective, even under a high speed condition.

**Figure 6:**
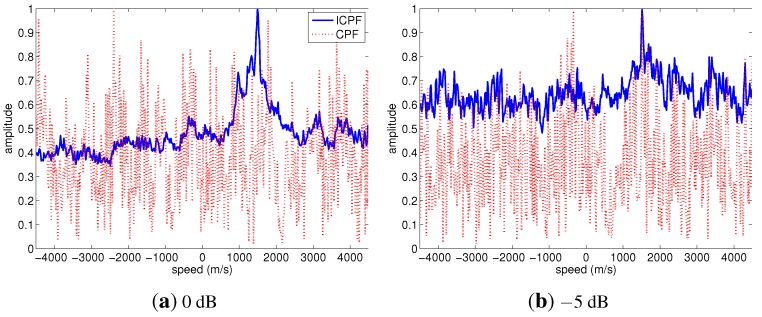
CPF and ICPF of the radar echo in different SNR conditions.

The performances of the speed estimation methods based on CPF and ICPF under low SNR conditions are considered. Figure 6 shows the CPF and ICPF of the radar echo under the conditions of SNR = 0 dB and SNR = 5 dB, respectively. It has been shown that the CPF curve is embedded in strong noise and does not exhibit a peak at a speed of 1500 m/s when SNR = 0 dB; while the ICPF is robust to noise, it still contains an obvious peak at 1500 m/s when the SNR is as low as −5 dB. Hence, the speed compensation method based on ICPF is more robust than that based on CPF.

**Figure 7:**
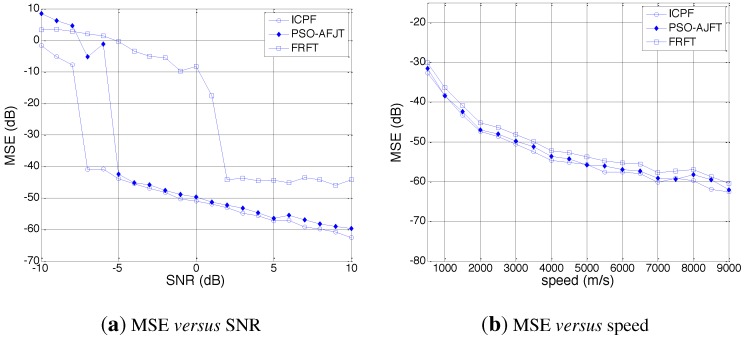
MSE for ICPF, particle swarm optimization-adaptive joint time-frequency (PSO-AJFT) and fractional Fourier transform (FRFT).

The performance of the proposed method is further compared to that of PSO-AJFT and FRFT.Figure 7 shows the Monte-Carlo simulation results for the speed estimation methods based on ICPF, PSO-AJFT and FRFT, where the number of Monte-Carlo simulations is set as 100. The mean squared error (MSE) for three methods in different SNR conditions is shown as Figure 7a, where MSE is defined as follows:

(24) $$MSE=10\text{lg}\frac{\sqrt{\frac{1}{N}\sum_{n=1}^{N}{\left[\hat{v}\left(n\right)-v\right]}^{2}}}{v}$$

where *N* is the number of Monte-Carlo simulations, $\hat{v}\left(n\right)$ is the speed estimated in the n-th simulation and *v* is the average speed. It can be seen that the MSE of ICPF is lower than that of PSO-AJFT and FRFT in any SNR condition. Moreover, when the SNR is as low as −7 dB, PSO-AJFT and FRFT are invalid, while ICPF still can estimate speed precisely.

Figure 7b shows the MSE *versus* speed of the target of the three methods. It can be seen that the MSE of ICPF is lower than that of PSO-AJFT and FRFT at any speed, which further verifies the effectiveness of the proposed method.

### 4.2. Experiment Based on the Radar Data of an Aircraft

**Figure 8:**
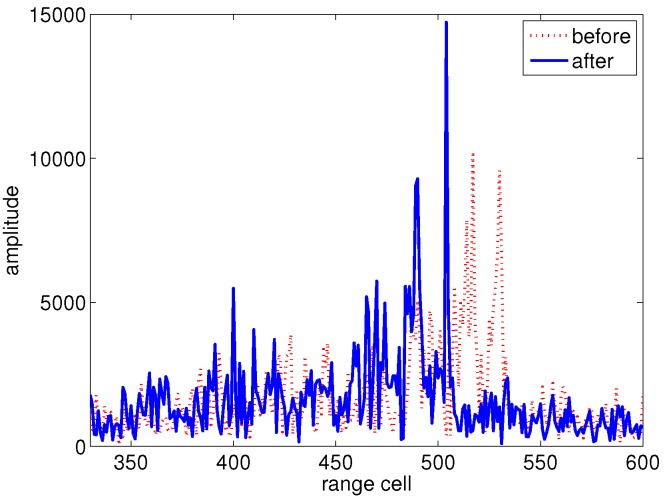
HRRP of the aircraft before and after speed compensation.

**Figure 9:**
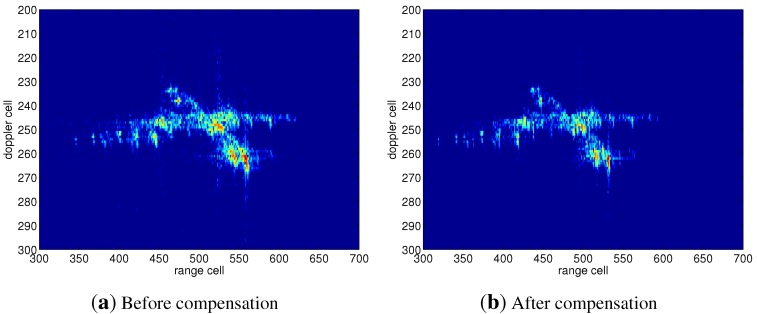
ISAR of the aircraft before and after speed compensation.

Radar data of an aircraft are utilized to further validate the effectiveness of the proposed methods. The waveform parameters are as follows: the center frequency is 9 GHz; the band width is 1 GHz; and the PRF is 250 Hz. Figure 8 shows the comparison between the HRRP before and after the speed compensation, where the dashed and real curves represent the HRRP before and after speed compensation, respectively. It has been shown that the HRRP after speed compensation is better focused than that before compensation. The ISAR images before and after speed compensation are shown as Figure 9. It can be seen from Figure 9a that the scatterers on the wing and tail of the aircraft are better focused than those shown in Figure 9b, which verifies the effectiveness of the proposed method.

**Figure 10:**
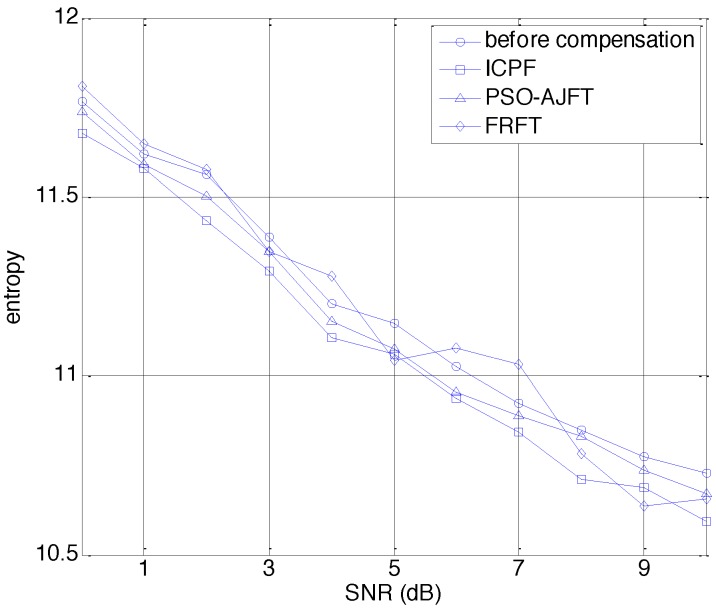
ISAR image entropy *versus* SNR for different methods.

Moreover, the image entropy [14] has been introduced to numerically compare the performance of ICPF with that of PSO-AJFT and FRFT. The smaller the image entropy is, the better the speed compensation method performs. Figure 10 shows the image entropy achieved by the three methods in different SNR conditions. It can be seen that the entropy obtained by ICPF is smaller than that achieved by PSO-AJFT and FRFT. Hence, ICPF compensates the speed more precisely than the other two methods.

## 5. Conclusions

Two novel speed compensation methods for ISAR imaging have been proposed in this paper, which are based on CPF and ICPF, respectively. The CPF-based method has the advantage in realization, but it is effected by cross-terms and not robust enough to satisfy the requirement of ISAR imaging in a low SNR condition. The ICPF-based method is more robust to noise and can precisely estimate speed, even when the SNR is as low as −7 dB. Experimental results based on measured data have validated the performance of the proposed algorithm.
